# Advancements in First-Line Treatment of Metastatic Bladder Cancer: EV-302 and Checkmate-901 Insights and Future Directions

**DOI:** 10.3390/cancers16132398

**Published:** 2024-06-29

**Authors:** Vijay Kumar Srinivasalu, Debbie Robbrecht

**Affiliations:** 1 Department of Medical Oncology, Pantai Jerudong Specialist Center, The Brunei Cancer Center, Jerudong BG3122, Brunei; 2 Department of Uro-Oncology, Erasmus MC Cancer Institute, 3015 CN Rotterdam, The Netherlands

**Keywords:** advanced bladder cancer, antibody-drug conjugates, novel therapies, immunotherapy

## Abstract

**Simple Summary:**

Bladder cancer represents a significant global health burden, particularly affecting elderly males. Incidence rates vary globally, with higher rates observed in western Europe than in South Asia. Metastatic bladder cancer has historically had poor outcomes, with platinum-based chemotherapy as the standard first-line treatment. In this review, we delve into the two pivotal trials, Checkmate-901 and EV-302, that addressed the need for improved first-line treatments and resulted in a paradigm shift with the incorporation of antibody–drug conjugates (ADCs) and immunotherapy combinations in the advanced bladder cancer conundrums. We also provide a comprehensive review of the novel therapeutic approaches, including fibroblast growth factor receptor inhibitors (FGFR), human epidermal growth factor receptor (HER) pathway inhibitors, and various other ADCs targeting antigens, such as nectin-4, Trop-2, and tissue factors that are available to improve the survival of advanced bladder cancer.

**Abstract:**

Advanced bladder cancer patients have historically failed to achieve prolonged duration of response to conventional chemotherapy and needed better first-line treatment regimens. The approval of nivolumab in combination with gemcitabine and cisplatin and pembrolizumab with antibody–drug conjugate enfortumab vedotin has revolutionized the first-line treatment of advanced bladder cancer in many countries. In this review, we summarize the intricate differences between the two landmark clinical trials that led to their incorporation into the current standard of care for advanced bladder cancer. We further discuss newer novel treatment options in the second and subsequent lines of treatment on progression, like immunotherapy in combination with other agents, including fibroblast growth factors receptor inhibitors, human epidermal growth factor inhibitors, antibody–drug conjugates, tyrosine kinase inhibitors, and novel antibodies. Finally, we discuss the integration of these novel therapies into current clinical practice amidst the rapidly evolving landscape of advanced bladder cancer treatment, aiming to enhance patient outcomes.

## 1. The Burden

Bladder cancer is one of the top 10 cancers in the world, contributing around 573,000 new cases and resulting in 213,000 deaths as per GLOBACAN 2020. It is a disease that is most prevalent in elderly males, with more than nearly one-third of them being over the age of 65 years at the time of diagnosis [1,2,3]. The incidence rate is 9.5 per 100,000, and the mortality rate is 2.3 per 100,000 among men [4]. The age-standardized incidence rates of bladder cancer are highest in western Europe (14.9 [95% UI 12.8 to 17.3]), followed by central Europe (12.6 [11 to 14.3]) and North Africa and the Middle East (9.6 [8.1 to 11.4]), and the lowest age-standardized incidence rates are in South Asia (2.4 [2.1 to 2.7]), Oceania (2.5 [2 to 3.1]), and Andean Latin America (2.5 [2.1 to 3.1]) as per the 2019 Global Disease Burden Study systematic analysis [5]. The GLOBOCAN 2020 factsheet shows a similar trend in the age-standardized incidence of bladder cancer, with the highest incidence from southern Europe (15.3 per 100,000) and the lowest age-standardized incidence from south–central Asia (1.9 per 100,000) and Middle Africa (1.6 per 100,000) [6]. This disparity in the incidence rates globally could be explained by the significant variability in access to tobacco, the extent of exposure to carcinogens, and the vast degree of industrial development [2,7].

The most common histological subtype of bladder cancer is urothelial carcinoma, contributing 90% in the United States and Europe [4]. Superficial or non-muscle-invasive (NMIBC) and muscle-invasive bladder cancer (MIBC) are the two most common types of bladder cancer [8], and at the time of diagnosis, 11% of patients have locally advanced or metastatic disease [9,10].

Tumors arising from the epithelial lining of the urinary tract from the renal calyces to the ureteric orifices are called upper urinary tract urothelial carcinoma (UTUC) [11]. UTUC is more prevalent in Asian countries [12] and contributes only about 5% of all urinary tract (including bladder) cancers [13]. In Taiwan, an increased incidence of UTUC was linked to exposure to aristolochic acid, a nephrotoxin carcinogen known to induce mutagenesis [14].

Metastatic bladder cancers (BCs) pose a significant therapeutic challenge, marked by dismal outcomes with conventional chemotherapy, yielding 5-year survival rates of a mere 7–10% [15,16]. These outcomes improved further with the advent of checkpoint inhibitors in the second-line or maintenance setting [17]. Until recently, the standard of care for first-line treatment of patients with metastatic BC who are willing for treatment has been platinum-based chemotherapy. Either cisplatin or carboplatin was combined with gemcitabine (GC) or methotrexate, vinblastine, doxorubicin, and cisplatin (MVAC) either as standard dose Q4 weekly or dose-dense Q2 weekly. In patients not eligible for cisplatin-based chemotherapy, carboplatin-based chemotherapy is considered along with gemcitabine. In patients without evidence of disease progression after 4–6 cycles, a sequential avelumab maintenance therapy was shown to improve overall survival. If ineligible for platinum-based chemotherapy (either cisplatin or carboplatin), single-agent immune checkpoint inhibitors are considered.

There has been a dire need to identify newer regimens in bladder cancer that induce durable, long-standing responses and improve survival outcomes, as platinum-based chemotherapy is not sustainable [18]. In this review, we analyze the newer standard-of-care options for the treatment of first-line metastatic bladder cancer patients and review the novel therapeutic options like fibroblast growth factor receptor (FGFR) inhibitors, Her2 inhibitors, TGF-beta inhibitors, and newer antibody–drug conjugates (ADCs) in the pipeline to be considered in the subsequent lines of treatment post-progression.

## 2. The Newer Standard of Care in First-Line Treatment of Metastatic Bladder Cancer Conundrum EV-302 and Checkmate-901

For over two decades, nothing new or innovative has been added to the existing treatment armamentarium that has resulted in an improvement in overall survival in the first-line treatment of advanced bladder cancer except for avelumab in the sequential maintenance setting for patients without evidence of disease progression with platinum-based chemotherapy [19]. This unmet need was addressed by two pivotal trials, Checkmate-901 and EV 302, presented at the ESMO-2023 plenary session.

Checkmate-901, a phase 3 randomized study that juxtaposed the conventional gemcitabine/cisplatin with the combination of nivolumab (anti-PD1) with gemcitabine/cisplatin for previously untreated, unresectable, or metastatic cisplatin-eligible bladder carcinoma, met its primary endpoint with a rather modest median overall survival (OS) enhancement favoring the nivolumab combination arm (21.7 vs. 18.9 months, HR: 0.75, 95% CI: 0.63–0.96; *p* = 0.017) [20]. The experimental arm of chemo-IO (chemotherapy with immunotherapy) had an overall response rate (ORR) of 58% with 22% complete response (CR) in comparison to 43% ORR and 12% CR with cisplatin-based chemotherapy alone. The duration of response was over 3 years (37.1 months) in the complete responder’s subgroup in the chemo-IO arm. The differences in the clinical outcomes between EV-302 and Checkmate-901 are tabulated in Table 1.

The EV-302 combined the anti-nectin-4/monomethyl auristatin E antibody–drug conjugate (ADC) enfortumab vedotin (EV) with anti-PD1 pembrolizumab (EVP) versus platinum-based chemotherapy (either cisplatin- or carboplatin-based) and demonstrated a staggering improvement in the median overall survival to 31.5 months (almost twice that of the comparator arm, 16.1 months) (HR, 0.47; *p* < 0.00001), with an overall response rate of 68% and nearly 30% of patients having a complete response compared to 44% ORR and 12% CR in patients receiving the platinum-based chemotherapy [21]. This is by far the highest overall response ever documented in a phase III trial treating advanced bladder cancer in the first-line setting, irrespective of the PDL-1 status.

“First hit is your best hit”, with the majority of the patients with advanced bladder cancer not being fit for a subsequent line of treatment due to poor general condition or compounding co-morbidities in the aging frail patients with advanced disease [22]. Studies in the pre-avelumab maintenance era have shown that nearly 60–70% of patients were not fit for second-line chemotherapy after progression on first-line treatments [23,24,25], which reemphasizes the need to choose an optimal first-line treatment regime. Currently, three options are in place in the 1L setting, though availability is different on a global scale: platinum-based chemotherapy followed by maintenance avelumab, EVP, or cisplatin/gemcitabine/nivolumab. It is at the discretion of the clinician to choose one of them wisely.

However, we should also place results from EV-302 in the context of the OS analyses used, which was different in EV-302 than in the Javelin Bladder 100 study with an updated median OS of 29.7 months measured from the start of first-line chemotherapy backed by real-world data [26,27,28], which is comparable to what has been done for OS analysis in EV-302, and not so different from the 31.5 months observed in EV-302. However, these survival results from the Javelin Bladder 100 study are prone to a selection bias since this represents a population that had benefited from their first-line chemotherapy.

Numerically, in the cross-trial comparison (although not ideal), the EV-302 has a higher apparent benefit in terms of doubling progression-free survival (PFS), ORR, and CR. However, nearly fifty percent received carboplatin-based chemotherapy due to cisplatin ineligibility, which could result in slightly inferior outcomes in the chemotherapy treatment arm.

IMVigor 130 [29] and KEYNOTE-361 [30] trials, combining checkpoint inhibitors with chemotherapy, did not meet their primary endpoints. In contrast, Checkmate-901 has been the only positive trial with combined therapy in this area, though patients had to be eligible for cisplatin. Cisplatin-based treatment induces transcriptional changes in circulating immune cells, resulting in increased antigen presentation and T-cell activation and culminating in more potent immunogenic cell death when compared to carboplatin [31]. On the other hand, in IMVigor 130, a contemporary large population, OS was the same in the carboplatin-treated population as in the cisplatin-treated population. Carboplatin or cisplatin was the decision of the treating physician. The retrospective evaluation showed that 75% of patients would have been eligible for cisplatin based on criteria [32], but only one-third of the patients were treated with cisplatin. So, in an unselected patient population, carboplatin might provide benefits equal to cisplatin. The observation of poorer outcomes with carboplatin in other trials might have just been a result of selection bias, as patients who were not fit for cisplatin were treated with carboplatin [33].

The attractiveness of Checkmate-901, specifically in cisplatin-eligible patients with suitable performance status, lies in its manageable toxicity profile, a finite number of cycles (4 or 6), and reduced costs (compared to EV-302), especially in low- and middle-income countries (LMICs) with lack of access to novel ADCs or avelumab.

**Figure 1:**
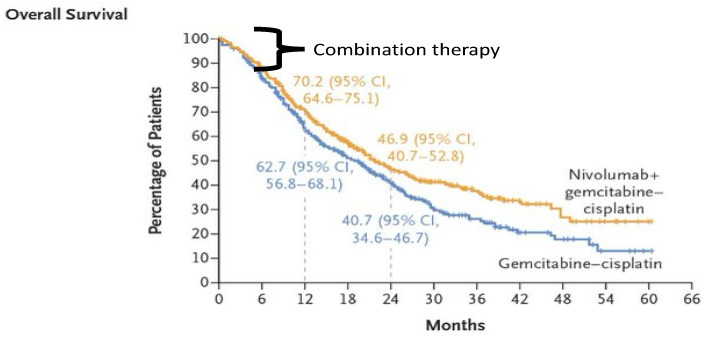
Overall survival curves of Checkmate-901 trial.

The three major criticisms of the study that contributed to a mere 12-week improvement in overall survival were the lack of transparency of the data with only subgroup analysis (arm-B) of the study being reported with no update on the primary study (arm-A) of the Checkmate-901 presented at ESMO 2020 [34] and a press release in May 2022 expressing a disappointment in not meeting the primary endpoint of overall survival [35]; the second criticism is the overlapping of survival curves with both the arms remaining together for over the initial 6 months with no clear separation indicating lack of synergism, and the maximum benefit seems to be driven by the nivolumab maintenance arm (Figure 1). The third is the inferior ORR of 43% demonstrated in the experimental arm, in comparison with the historical ORR of around 70% in the Javelin Bladder 100 trial, irrespective of the PD-L1 status.

The identification of potential biomarkers of complete responses in the metastatic setting, like inherent platinum sensitivity with HRR pathway deficiency or ERCC2 mutations (as seen in the neoadjuvant setting), would aid in more effective patient selection.

The subgroup analysis of EV-302 favored the experimental arm of EVP in both cisplatin-eligible and cisplatin-ineligible patients, irrespective of the level of PDL-1 expression [36]. The ADC-IO combination of EVP is perhaps more appropriate for the first-line treatment of advanced bladder cancer patients with platinum ineligibility and a slightly preserved performance status. However, close consideration is warranted due to the occurrences of unique toxicities like peripheral neuropathy, ocular symptoms, skin reactions that are likely linked to nectin-4 expression in the skin, and less common severe hyperglycemia in more than half of the treated patients, with over 20% of the patients discontinuing enfortumab vedotin due to adverse events. On assessing the cost-effectiveness of EV, it is highly priced, with 1 cycle of EV being equivalent to 127 cycles of gemcitabine-based chemotherapy as per the article in European Urology. With the median number of cycles received placed around 12 cycles, this combination would not be easily accessible to most low- and middle-income countries with stringent reimbursement policies, regulatory authorities, and health systems trying to reduce the ever-increasing financial toxicity.

As responders continue to remain on the EVP regime until progression or toxicity while the IO is stopped at 2 years, an alternative strategy of dose de-escalation or drug holiday would help mitigate the toxicity and reduce the costs of treatment. Another major pitfall is the median duration of follow-up being very short, only 17 months. With the majority of patients being censored for survival, the hazard ratio (HR) is most likely to change with a longer duration of follow-up. The comparator arm of EV-302 has slightly underperformed when compared to the gemcitabine–cisplatin combination in Checkmate-901 (16.1 months vs. 18.9 months, although this is a cisplatin-eligible versus cisplatin-ineligible subgroup comparison). This study also does not represent the real-world usage of ADCs post-chemotherapy and post-PDL-1 inhibitor, as EV monotherapy was already approved following the EV-301 study, which offered a 30% lower risk of death versus investigators of choice chemotherapy. Only 3.8% of patients on the comparator chemotherapy arm in the trial received ADCs on progression, while in the real world, up to 60% of patients received EV as second-line treatment following avelumab maintenance [37].

## 3. Novel Treatment Options for Advanced Bladder Cancer

Identification of novel biomarkers for response to EV is needed, especially since the level of nectin-4 expression seems not to be advantageous [38]. Evaluation of pre- and on-treatment tumor samples would shed light on inherent and acquired resistance mechanisms.

A major challenge is what to do in patients with progressive disease on first-line EVP, taking into account that progression might occur during the EVP combination but also during EV monotherapy or pembrolizumab monotherapy, when the other part of treatment has been discontinued, for example, due to toxicity. This poses several challenging questions, particularly when an actionable target like FGFR2/3 alterations and Her-2 expression is lacking. Thanks to significant progress in the understanding of the molecular and genomic profile of bladder cancer, its signaling pathways and genetic alterations that drive bladder cancer progression have been uncovered. These advances have resulted in the identification of novel targets that are actionable, like FGFR inhibitors, Her2 inhibitors, drugs targeting TGF-beta, newer ADCs, etc. (Table 2).

A. Fibroblast Growth Factor (FGF) Receptor

Five receptors in the FGFR family (FGFR1–4 and FGFRL1) play an important role in cell proliferation, angiogenesis, differentiation, metabolism, motility, and invasion. The binding of the FGF to its receptor would result in the phosphorylation of proteins and activation of the Ras/Raf/MEK-MAPK pathway, PI3K/AKT pathway, PLCγ, and signal transducer and activator of transcription (STAT) downstream signaling cascades [39,40]. Among the five FGF receptors, FGFR3 activating missense mutations and in-frame FGFR3-TACC3 fusions are the most common alterations found in nearly 20% of advanced bladder cancer [41,42,43]. These aberrant alterations, including fusions, amplifications, and mutations, contribute to the proliferation, metastasis, and drug resistance in cancer cells [44].

Tumors harboring FGFR3-TACC3 fusions and FGFR3-hyperactivated mutations exhibit an excellent response to FGFR3 inhibition [45,46,47,48]. These receptors could be inhibited by small-molecule tyrosine kinase inhibitors (TKI) or neutralizing antibodies and traps of FGF ligands that target FGFR signaling. The novel small-molecule TKI with approved access in clinics is erdafitinib, used in the treatment of advanced or metastatic urothelial cancers that harbor an FGFR3 genetic alteration in the second line on progression after both platinum-based chemotherapy and PD-1 or PD-L1 inhibitors.

Early clinical studies with erdafitinib, an oral TKI that inhibits all four FGFR proteins, induced a response rate of 40% and median overall survival of 11 months [49] and led to an accelerated FDA approval for the second-line treatment. Based on these data, the THOR (cohort 1) phase III open-labeled trial compared erdafitinib against the investigator’s choice of chemotherapy (docetaxel or vinflunine) in patients with FGFR2- and FGFR3-altered advanced bladder cancer who progressed on one or two lines of chemotherapy, including a PD-1 or PD-L1 inhibitor, and resulted in a 36% reduction in the risk of death and 42% reduction in the risk of progression with manageable toxicities [50]. Erdafitinib was also compared with pembrolizumab in patients with FGFR2 and FGFR3 alteration who progressed on one prior therapy and immunotherapy naïve in the THOR (cohort2) study and demonstrated a similar overall survival of 11 months in both arms at a median follow-up of 33 months [51].

Infigratinib is a potent FGFR1–3 selective oral TKI, granted FDA approval based on the phase II data that reduced the size of the tumors with a disease control rate of 64.2% in advanced bladder cancers that harbor FGFR3 genetic alterations [52,53].

Resistance to FGFR inhibitors has resulted in the designing of various alternate strategies by combining erdafitinib with enfortumab vedotin in a phase 1b study. The study was feasible, with preliminary antitumor activity in all eight recruited patients [54]. FGFR3-mutated advanced bladder cancer exhibits a low inflammatory signature [41] with lower response rates to immune checkpoint inhibitors [55]. On the contrary, erdafitinib, in combination with immune checkpoint inhibitors, has contributed to increased TCR clonality and decreased tumor-associated macrophages [56]. Hence, numerous early-phase trials are combining FGFR inhibitors with anti-PD1/PD-L1 tumors to counteract resistance and achieve sustained, durable responses.

Vofatamab is a monoclonal antibody that targets the extracellular domain of FGFR3 and blocks the binding of ligands to cognate FGF receptors, thus preventing receptor signaling [57]. In FIERCE-21, a phase Ib/II study, advanced bladder cancer patients who progressed on platinum-based chemotherapy were treated with vofatamab alone or in combination with docetaxel. A proportion of patients benefited from the combination treatment [58]. Early-phase clinical trials show promising activity when vofatamab is combined with pembrolizumab in platinum-refractory bladder cancer [59]. LOX0-435 is a phase 1 study exploring its safety and efficacy in advanced solid tumors, including bladder cancer with FGFR3 alteration (NCT05614739).

B. Human Epidermal Growth Factor (HER) receptor

Represented by EGFR (ErbB1), HER2 (ErbB2), HER3 (ErbB3), and HER4 (ErbB4), these receptors on activation cause downstream activation of the signaling cascade, resulting in cell growth, proliferation, and possibly resistance to chemotherapeutic agents [60,61]. Among the EGFR receptors, HER2 is unique, as it can generate downstream signals without a ligand, whereas other receptors require binding to a ligand for homo- or heterodimerization of the receptor to produce downstream effects. The incidence of HER family genomic alterations like mutation, amplification, and overexpression are noted in around 20–30% of patients with bladder cancer, with The Cancer Genome Atlas (TCGA) and other datasets showing a similar concordance [41,62,63,64].

The co-relation of HER2 expression with clinical outcomes is mixed [60,65,66,67], with one possible pitfall for clinical translation of HER2 as both a prognostic or predictive biomarker is the discordance between HER2 fluorescence in situ hybridization (FISH), immunohistochemistry (IHC) expression, and genomic-level molecular characterization. There is no FDA-approved HER2-targeted therapy in bladder cancer. Some monoclonal antibodies like trastuzumab, small-molecule tyrosine kinase inhibitors like lapatinib, afatinib, and neratinib, and antibody–drug conjugates like trastuzumab emtansine (TDM-1), trastuzumab deruxtecan (TDX-d), and fam-trastuzumab deruxtecan-nxki are being explored in early clinical trials.

Trastuzumab, when combined with chemotherapy in a phase II clinical trial, resulted in an improved median disease-free survival of 9.3 months and overall survival of 14.1 months in patients with HER-2-positive advanced bladder cancer [68]. Lapatinib was explored in the second-line treatment of advanced bladder cancer with EGFR or HER-2 overexpression and resulted in a median overall survival of 17.9 weeks [69]. Similarly, afatinib showed promising results in patients with platinum-refractory bladder cancer harboring HER2 or HER3 mutations in a phase II study [70]. A study on neratinib in patients with advanced bladder cancer with a HER2-GRB7 gene fusion is underway.

Disitamab vedotin, an ADC-targeting HER-2, significantly improved outcomes in patients with HER-2-expressing advanced bladder cancer with an overall response rate of 51.2% and median overall survival of 13.9 months [71]. The FDA granted a “Breakthrough Therapy Designation” based on this study. Combining disitamab vedotin with tremelimumab in patients with no prior treatment in first-line metastatic bladder cancer resulted in an overall response rate of 80%, with the ORR based on the IHC as 100% for HER2-3+, 77.8% for HER2-2+, 66.7% for HER2-1+, and 50% for HER2-negative cases [72].

Trastuzumab emtansine (TDM-1) has shown promise in HER-2-positive bladder cancer cell lines [73], with trastuzumab deruxtecan (TDX-d) preliminary results showing promise in combination with nivolumab in second and later lines of treatment in HER-2-expressing bladder cancer. DESTINY-PanTumor02 trial showed TDX-d to elicit clinically meaningful activity in all advanced solids humors harboring HER-2 alteration, including bladder cancer [74], and has received a priority review designation by the FDA [75]. In patients with the HER-2 IHC-3+ subgroup, the ORR was 61.3%, with a median PFS and OS of 11.9 and 21.1 months, respectively [76]. Recently, the FDA granted accelerated approval to fam-trastuzumab deruxtecan-nxki for patients with unresectable and metastatic HER2-positive (IHC-3+) solid tumors (including bladder cancer) who have received prior systemic treatment and have no satisfactory alternative treatment options [77].

C. Antibody–Drug Conjugates (ADC)

  a. Sacituzumab govitecan (SG)

It is an ADC that targets Trop-2, a transmembrane glycoprotein that binds to an active metabolite of Irinotecan, SN-38. This drug is approved by the FDA for patients with advanced bladder cancer following progression on both platinum-based chemotherapy and PD-1 or PD-L1 inhibitor [78] following the TROPHY-U-01 phase II study with an ORR of 27% and a median overall survival and progression-free survival of 11 and 5 months, respectively, in platinum and immune checkpoint inhibitor refractory population [79]. Higher Trop2 expression levels in the EV-resistant cell lines [80] imply that sacituzumab govitecan (SG), an ADC that targets Trop-2, may be a potential alternative. Despite the downregulation of nectin-4 following extended EV exposure, cell lines’ sensitivity to Trop2 was well maintained, indicating that the two ADCs’ resistance mechanisms did not overlap. However, a clinical trial in the EV refractory population and post-EVP second-line setting is urgently needed.

  b. Datopotamab deruxtecan (Dato-DXd)

It is an ADC of humanized anti-TROP2 IgG1 monoclonal antibody that has shown promising antitumor activity with a manageable safety profile in patients with solid tumors, including bladder cancer. In patients with advanced bladder cancer who progressed on more than one line of treatment, the ORR was 27.8%, with a disease control rate of 77.8%, with tumor reduction in 82% of the participants [81]. Most were post-EV treatment and serve as an essential salvage regimen and are being explored further in the phase 1/2 TROPION-PanTumor02 and the phase 2 TROPION-PanTumor03 studies.

  c. Sirtratumab vedotin

This ADCs target SLITRK6 (SLIT and NTRK-like protein 6 is a protein that in humans is encoded by the *SLITRK6* gene), which is commonly expressed in almost 88% of BC specimens on tissue microarrays. This ADC delivers the monomethyl auristatin E to cells with SLITRK6 expression by connecting specific antibodies via a protease-cleavable linker [82]. A phase 1 study showed the drug sirtratumab vedotin to be active with an ORR of 33%, with promising activity in advanced BC patients with previous treatment failure on immune checkpoint inhibitors [83].

  d. Tisotumab vedotin

ADCs that target tissue factor (TF). This transmembrane glycoprotein plays a vital role in cancer angiogenesis, growth, and metastasis and acts as an initiator of TF in the coagulation pathway [84]. The first in-human ADC, tisotumab vedotin, in early phase I/II clinical trials has shown preliminary anticancer activity with an ORR of 26.7% in BC [85]. Targeting TF with immunotherapy is promising, with potential efficacy and safety, and needs to be explored in the future.

Understanding the mechanisms of resistance to ADCs, like different signaling pathways, alterations in the payload efficiency, processing and internalization, and blockade of antibody attachment [86], would help in designing better ADCs with reduced off-target toxicity. A list of all the ADCs with promising clinical benefits in advanced BC is summarized in Table 3.

D. Vascular endothelial growth factors receptor (VEGFR)

Several studies have shown that inhibition of VEGF transcripts contributed to decreased proliferation rate, reduced growth, and invasion in BC cells [87,88,89]. Sunitinib and pazopanib are VEGF TKIs tested in the second-line or first-line platinum-ineligible patients with advanced BC and demonstrated lower response rates, low accrual, and toxicity. A newer TKI, cabozantinib targets VEGFR, MET, AXL, and RET and has higher activity than other TKIs in advanced urothelial carcinoma [90].

  a. Cabozantinib

The COSMIC-021 study combined cabozantinib with atezolizumab in advanced urothelial cancer with no prior systemic treatment in both cisplatin-ineligible (cohort 3) and cisplatin-eligible patients (cohort 4) with promising disease control rates of 80% and 63%, respectively. But the caveats for this study were a small number, and the duration of response was lower than that reported in IMVigor 210 with single-agent atezolizumab. In patients with prior immune checkpoint-treated advanced BC cohort in the COSMIC-021 study, ORR was 10% and not that different from responses with cabozantinib and nivolumab combination (ORR of 16%). It can be concluded that cabozantinib does not seem to be adding much benefit. The combination of cabozantinib plus avelumab is being evaluated in the phase III MAINCAV study [91]. The MAINCAV study compares maintenance cabozantinib and avelumab versus maintenance avelumab in patients with durable response to 1L platinum-based chemotherapy in metastatic BC.

  b. Famitinib

It targets the stem cell factor receptor (c-kit), VEGFR-2, and PDGFR*β*, and it has anti-angiogenesis and antitumor cell proliferation activity. A phase II clinical trial combined famitinib with camrelizumab, a monoclonal antibody against PD-1, and achieved an objective efficacy rate of 38.9% and a median PFS time of 8.3 months in patients with advanced or metastatic BC after platinum-based chemotherapy [92].

Sorafenib was explored in a phase I clinical trial by combining it with a microtubule inhibitor. Vinflunine was found to be safe and efficacious and achieved an ORR of 41% in second-line therapy after the progression of platinum-based chemotherapy in metastatic BC [93].

The treatment landscape for metastatic bladder cancer is evolving dynamically with the global variability in accessibility to new treatment options, leading to the heterogeneous use of EVP and nivolumab/gemcitabine/cisplatin combinations. However, with the conceptual evolutionary change in oncology as drugs are cycled from metastatic to adjuvant and neoadjuvant setting, the approvals of adjuvant checkpoint inhibitor nivolumab in high-risk muscle-invasive bladder cancer post-radical cystectomy (Checkmate-274) [94], pembrolizumab in the BCG-refractory non-muscle-invasive setting [95], and the combination of EV+P is being explored in the perioperative setting versus gemcitabine + cisplatin in cisplatin-eligible MIBC [96] and also similarly in cisplatin-ineligible MIBC in the perioperative setting [97], together adds to the complexity of choosing the next step at progression.

In contrast to combination chemotherapy, immunotherapy, ADCs, and TKI approaches, drugs targeting metabolic dysfunction and signaling pathways are making significant inroads in urothelial cancer, especially in early-stage disease. With advancements in high-throughput screening technologies, including transcriptomics, metabolomics, and proteomics, screening for targets is currently an important research focus. Identification of specific markers of bladder cancer stem cells (BCSCs) could help design corresponding targeted drugs, as these BCSCs contribute to significant drug resistance induced by chemotherapy. Designing clinical trials with combination therapies targeting different pathways, including signaling pathways or metabolic pathways, could be promising in improving our survival outcomes for advanced bladder cancer.

## 4. Conclusions

In the era of EV-302 and Checkmate-901, there is still a definite role in day-to-day practice for the Javelin Bladder 100 kind of regimen with sequential immunotherapy maintenance following platinum-based chemotherapy in responders. It is important to consider the various clinical parameters, performance status, reimbursement policies, accessibility to these drugs, and patient considerations before treatment initiation and encourage enrolment into clinical trials whenever an opportunity arises to understand the biology of the disease and identify a subset of patients who seem to derive the maximum benefit through these strategies. Overall, the evolving landscape of first-line treatments for metastatic bladder cancer offers new hope for patients, with a focus on improving survival outcomes and minimizing treatment-related toxicities.

## Figures and Tables

**Table 1:** The differences in the clinical outcomes between EV-302 and Checkmate-901.

Clinical Variables	EV-302	CHECKMATE-901
Regimen Used	Enfortumab Vedotin + Pembrolizumab	Gemcitabine + Cisplatin + Pembrolizumab
Comparator Arm	Gemcitabine + Cisplatin or Carboplatin included	Gemcitabine + Cisplatin only
Median PFS	12.5 vs. 6.3 months (HR: 0.45, *p* < 0.00001)	7.9 vs. 7.6 months (HR: 0.72, *p* = 0.0012)
Median OS	31.5 vs. 16.1 months (HR: 0.47; *p* < 0.00001)	21.7 vs. 18.9 months (HR: 0.75, *p* = 0.017)
Overall Response Rate (ORR)	68% vs. 44%	58% vs. 43%
Complete Responses (CR)	30% vs. 12%	22% vs. 12%

**Table 2:** List of novel targeted therapies in advanced bladder cancer.

Molecular Targets	Target Site	Median Disease-Free Survival	Phase of Clinical Trial
1. Fibroblast growth factor inhibitors			
A. Erdafitinib	FGFR 1–4, Oral TKI	11 months	Phase III
B. Infigratinib	FGFR 1–3, Oral TKI	NA	Phase II
C. Vofatamab	FGFR3, Mab	NA	Phase Ib/II
D. LOXO-435	FGFR3, Oral TKI	NA	Phase I
2. Human epidermal growth factor receptor inhibitors			
A. Trastuzumab	Her-2	9.3 months	Phase II
B. Lapatinib	Her-2	4.1 months (mOS)	Phase II
C. Disitamab Vedotin	Her-2	13.9 months (mOS)	Phase II
D. Trastuzumab Emtansine (TDM-1)	Her-2	11.9 months	Phase II
E. Fam-trastuzumab deruxtecan	Her-2	NA	Phase II
3. Vascular endothelial growth factor receptor inhibitors			
A. Cabozantinib	VEGF, MET, AXL, and RET	NA	Phase II
B. Famotinib	VEGFR-2, PDGFR-Beta, c-kit	8.3 months	Phase II
C. Sorafenib	VEGFR	NA	Phase I

**Table 3:** List of ADCs with promising clinical benefits in advanced metastatic BC.

ADC of Interest	Target	Phase of Development	Clinical Benefit
Enfortumab Vedotin	Nectin-4	Phase III	ORR—68% Median PFS—12.5 months Median OS—31.5 months
Sacituzumab Govitecan	Trop-2	Phase II	ORR—27% Median PFS—5 months Median OS—11 months
Disitamab Vedotin	Her-2	Phase II	ORR—51.2% Median OS—13.9 months
Trastuzumab Deruxtecan (TDX-d)	Her-2	Phase II	ORR—61.3% Median PFS—11.9 months Median OS—21.1 months
Datopotamab Deruxtecan (Dato-DXd)	Trop-2 IgG1	Phase I	ORR—27.8%
Tisotumab Vedotin	TF (tissue factor)	Phase I/II	ORR—26.7%
Sirtratumab Vedotin	SLITRK6	Phase I	ORR—33%
